# Protective Effect of Soy Lecithin Liposomes in Oxazolone-Induced Dermatitis in a Mouse Model: A Comparative Evaluation

**DOI:** 10.5812/ijpr-170144

**Published:** 2026-08-08

**Authors:** Alireza Partoazar, Maryam Alemi, Partow Mirzaee Saffari, Maryam Eskandary Nasab, Tayebeh Rastegar, Ahmad Reza Dehpour, Ramin Goudarzi

**Affiliations:** 1 Experimental Medicine Research Center, Tehran University of Medical Sciences, Tehran, Iran; 2 Department of Anatomy, School of Medicine, Tehran University of Medical Sciences, Tehran, Iran; 3 Department of Pharmacology, School of Medicine, Tehran University of Medical Sciences, Tehran, Iran; 4 Division of Research and Development, Pharmin USA, LLC, San Jose, USA

**Keywords:** Eczema, Lecithin, Liposome, Inflammation, Animal Model

## Abstract

**Background:**

Atopic dermatitis (AD) is a chronic inflammatory skin disorder characterized by intense itching, severe erosion, and rashes on the skin surface. Prolonged AD may cause hemorrhage and secondary infections due to scratching.

**Objectives:**

This study aimed to investigate the anti-AD effects of soy lecithin (SL) applied topically to oxazolone (OX)-treated skin in a mouse model.

**Methods:**

The effects of SL and hydrocortisone (HC), used as the standard drug, on behavioral responses such as scratching and grooming were evaluated during a 35-day trial. Histopathology and immunohistochemistry (IHC) for the cytokines TNFα and IL8 were also evaluated in the skin of the treated mice.

**Results:**

Topical administration of SL showed efficacy comparable to that of HC and significantly alleviated itching and grooming behaviors in eczematous animals. Topical SL also improved symptoms, including skin erythema, scarring, and edema, as well as histopathological parameters in AD mice. IHC analysis indicated that SL administration effectively reduced tissue levels of the inflammatory cytokines TNFα and IL8 in AD mice.

**Conclusions:**

Topical SL significantly improved AD severity in an OX-induced mouse model of AD. These results support further preclinical studies before clinical investigations in humans are undertaken.

## 1. Background

Atopic dermatitis (AD), also referred to as eczema, is a chronic inflammatory skin disease that is commonly managed with drugs. AD is characterized by severe itching, a mild-to-severe rash, edema, and erosion of the skin surface (1, 2). Various immunological, environmental, and neuropsychological factors can influence the development of this condition. A major feature of eczema is the presence of inflammatory cells, including lymphocytes, eosinophils, and neutrophils, and their cytokines at lesion sites (3).

AD stimulants affect keratinocytes and trigger the release of cytokines such as TNFα, leading to the production of proinflammatory cytokines, including IL1β, IL6, and IL8, by other keratinocytes and dermal cells (4, 5). Among inflammatory cytokines, IL8 shows the strongest correlation with lesion severity scores in patients with AD. IL8 can contribute to the activation and regulation of various immune cells in inflamed tissues (5, 6). TNFα, another cytokine implicated in AD, decreases the levels of long-chain free fatty acids and ceramides, consequently affecting lipid organization and distribution and resulting in skin injury (4). Moreover, skin barrier dysfunction in AD facilitates the penetration of AD stimulants into the dermal layers, thereby triggering an immune response.

One of the main therapeutic options for eczema is topical corticosteroids such as hydrocortisone (HC) (7). However, prolonged use may lead to significant adverse effects, including hyperglycemia and dermal and epidermal injury (8). Consequently, the development of novel therapeutic agents remains a focus for improving AD treatment. In this context, natural products are promising candidates because of their safety and potential efficacy (9, 10).

Soybean lecithin compounds mainly consist of natural glycerophospholipids such as phosphatidylcholine (PC), phosphatidylserine (PS), and other phospholipids. Lecithin and phospholipids are widely used in the food, pharmaceutical, and cosmetic industries as solubilizers and emulsifiers, as well as in other pharmaceutic-nutraceutical applications (11, 12). Experimental and clinical reports have indicated that lecithins and essential phospholipids have pharmacological potential for the control of certain disorders (13, 14). For example, the anti-inflammatory and antioxidative properties of lecithins have been identified as beneficial for wound healing (15) and gastric diseases (16). Their administration has an overall effect on inhibiting inflammation through immune-cell activation, proliferation, and cytokine production (17). Liposomes prepared from lecithin compounds or pure phospholipids have been extensively developed for dermal delivery or therapy in skin disorders (18, 19). They have shown beneficial effects on immunomodulation and inflammation reduction (20, 21).

## 2. Objectives

This study aimed to evaluate the effects of soy lecithin on dermatological features and the expression of the inflammatory cytokines TNFα and IL8 associated with AD in mice.

## 3. Methods

### 3.1. Chemical and Drug Preparation

Oxazolone (OX) was purchased from Sigma-Aldrich (USA). Soy lecithin (SL) was obtained from Pharmin USA, LLC (USA). Topical 1% HC was obtained from Tosan Daru (Iran). Pure ethanol (99.8%) was obtained from Alcohol Pars Co (PJS) (Iran).

A 5% (w/v) OX solution was prepared in pure ethanol for AD induction on mouse skin. Topical SL liposomes were prepared based on our previously reported method for wound healing (15). Briefly, 5% (w/v) SL was stirred and hydrated with phosphate-buffered saline (PBS; pH 7.2) to obtain a liposomal suspension. The suspension was then homogenized for 10 minutes to prepare fine, monodisperse SL liposomes.

The mean diameter and surface charge of the liposomes were determined by dynamic light scattering (DLS) using a Zetasizer system (Malvern Instruments Ltd). Each suspension was also examined using a transmission electron microscopy (TEM) system (Zeiss LEO 906, Germany).

The final SL liposomal suspension was prepared at 5% (w/v). Each mouse received 100 µL of this suspension topically, corresponding to 5 mg of SL per mouse per day, on the shaved dorsal skin area (approximately 2 - 2.5 cm^2^).

### 3.2. Animals

Female BALB/c mice weighing 28 ± 5 g were obtained from Tehran University of Medical Sciences. The animals were acclimatized for 1 week before the experiment under controlled conditions of 23 ± 2 °C, 50% humidity, and a 12:12-hour dark/light cycle. The study protocol was approved by the Committee on Experimental Animal Ethics of Tehran University of Medical Sciences (No. IR.TUMS.MEDICINE.REC.1400.807).

### 3.3. Study Design and AD Induction

The sample size was determined based on historical data from our laboratory and previous studies (22, 23) to ensure adequate statistical power.

Animals were first stratified by body weight and then randomly allocated to 4 experimental groups (n = 4 mice per group), ensuring a balanced distribution of body weights among the groups: C (vehicle), ethanol plus PBS; OX (negative control), OX plus PBS; HC (positive control), OX plus 1% HC; and SL (intervention), OX plus SL. In this study, 5% (w/v) SL was applied based on its wound-healing efficacy reported in our previous work (15). HC was applied topically to compare its effect with that of SL in AD mice.

AD induction was performed according to the modified Goudarzi method (9) as follows. On day 0, animals were anesthetized with ketamine (90 mg/kg) and xylazine (9 mg/kg), and the hair on the upper back was shaved. From days 1 to 7, 10 μL of ethanol was applied topically to the shaved dorsal area in the vehicle group, whereas 10 μL of 5% (w/v) OX was applied in the other groups. Treatments were applied topically once daily at 60 rubs per minute to the shaved dorsal area. From days 7 to 35, 60 μL of ethanol was applied in the vehicle group, and 60 μL of 0.5% (w/v) OX was applied topically in the other groups.

Thirty minutes after each OX or ethanol application, 100 μL of PBS, HC, or SL was applied topically to the shaved dorsal area (approximately 2 - 2.5 cm^2^) once daily at 60 rubs per minute in all groups to ensure equivalent volume, handling, and application conditions. No animals were excluded, lost, or replaced during the experimental period.

### 3.4. AD Severity Scores (Primary Endpoint)

The AD severity score was estimated as the sum of the scores for excoriation/erosion, scarring/dryness, edema, and erythema/hemorrhage. These components were scored separately by 2 observers at the end of each week, and the final score for each mouse was calculated as the mean. Excoriation/erosion, scarring/dryness, and edema were scored as 0 (none), 1 (mild), 2 (moderate), and 3 (severe). Erythema/hemorrhage was scored as 0 (none), 1 (very slight [light pink]), 2 (well-defined [dark pink]), 3 (moderate to severe [light red]), and 4 (severe [dark red]) (24).

### 3.5. Behavioral Assessments (Secondary Endpoint)

Behavioral testing was conducted at the end of each week using acrylic cages with 4 cells measuring 15 cm × 10 cm × 10 cm. Each cell had small openings for air circulation. To acclimatize the animals to the test conditions, they were handled and repeatedly placed in the containers before the experimental day. Animal behaviors were recorded by a camera for 20 minutes in the absence of observers. The numbers of scratching and grooming reactions and the duration of licking were assessed separately by a blinded investigator using coded video files (25).

### 3.6. Histopathological and IHC Analysis (Exploratory/Mechanistic Endpoint)

One day after the end of the fifth week, the animals were euthanized with CO_2_. Upper dorsal skin samples were collected and fixed in 10% formalin (v/v in PBS). Tissues were dehydrated, embedded in paraffin, and sectioned at 5 μm. Hematoxylin and eosin (H&E) staining was performed for histopathological evaluation. All slides were randomly coded and examined blindly by an expert pathologist using a light microscope. For each mouse, the final scores were determined based on the average of multiple sections. Epidermal thickness was measured using ImageJ software (NIH, USA), and collagen deposition, leukocyte infiltration, and angiogenesis were assessed semiquantitatively and scored from 0 to 3 (0 = none, 1 = mild, 2 = moderate, and 3 = severe) (24). In addition, IHC staining was performed using TNFα and IL8 antibodies (Biorbyt, Cambridge, United Kingdom). Cytokine levels were assessed by blinded examination of coded slides by an expert pathologist under a light photomicroscope and were scored as follows: Negative (0 - 1), weak (2 - 3), moderate (4 - 6), and severe (7 - 8). The presence of brown cells on the slides indicated expression of the respective cytokine markers (26).

### 3.7. Statistical Analysis

Statistical analysis was performed using GraphPad Prism version 8. For weekly repeated measurements, ordinary one-way ANOVA followed by Dunnett's multiple comparison test was applied separately at each time point. For single-time-point outcomes, the same test was used. All data are presented as mean ± SEM. Normality and homogeneity of variances were assessed, and P < 0.05 was considered statistically significant.

## 4. Results

### 4.1. Characteristics of SL Liposomes

**Figure 1.**
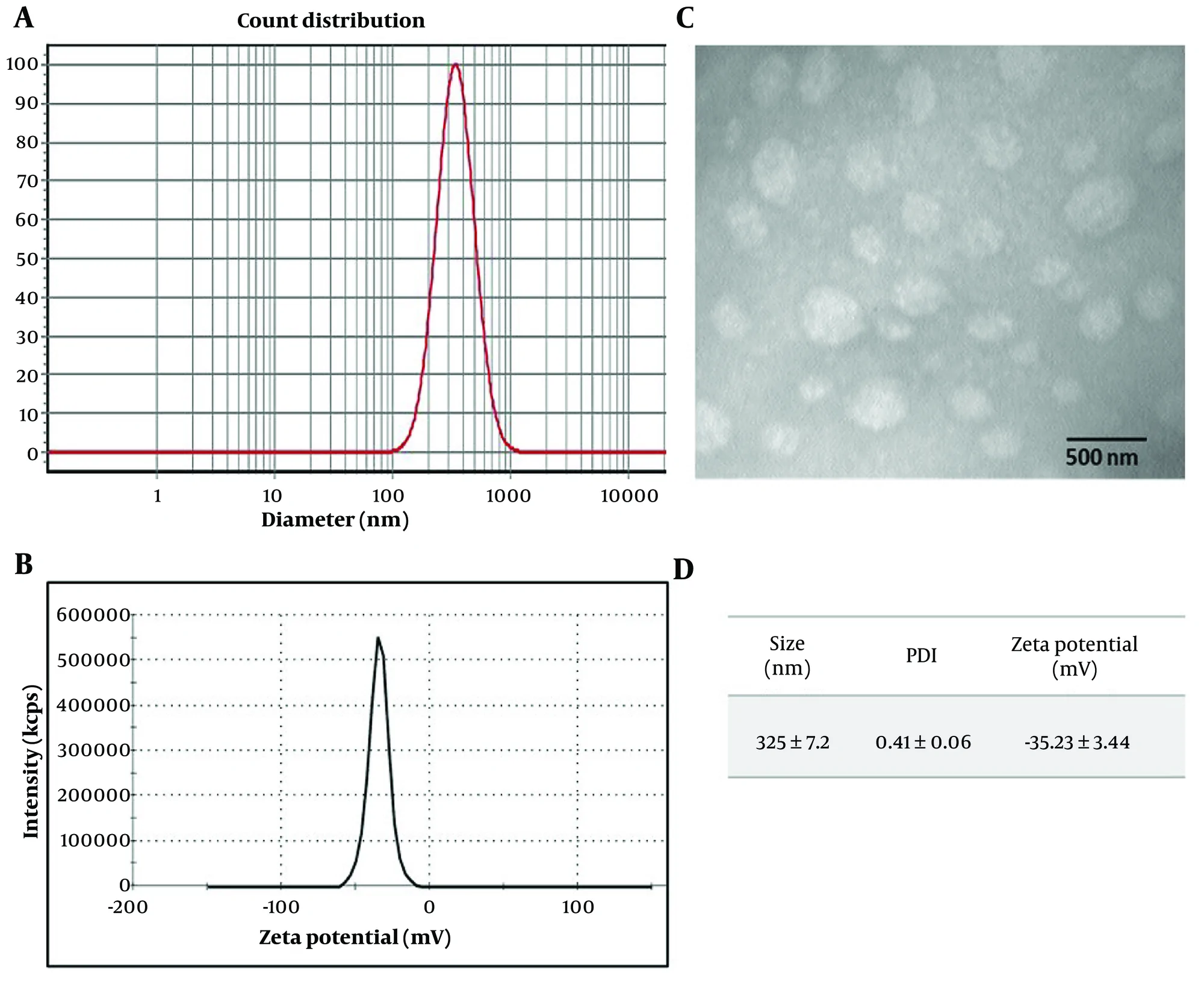
Representative characterization of SL liposomes: A, size; B, surface charge measured by DLS; C, photograph of the suspension obtained by TEM (50000×); and D, size, PDI, and zeta potential (surface charge) of SL liposomes (n = 3).

As shown in Figure 1, the average size of SL liposomes was approximately 325 nm, with a polydispersity index (PDI) of 0.41 ± 0.06, as determined by DLS analysis. The zeta potential of the SL vesicles indicated a negative surface charge (-35.23 ± 3.44), approximately -35 mV (Figure 1B and D). Microscopic evaluation of the SL suspensions confirmed the formation of spherical liposomes by TEM (Figure 1C).

### 4.2. AD Severity Scores (Primary Outcome)

**Figure 2.**
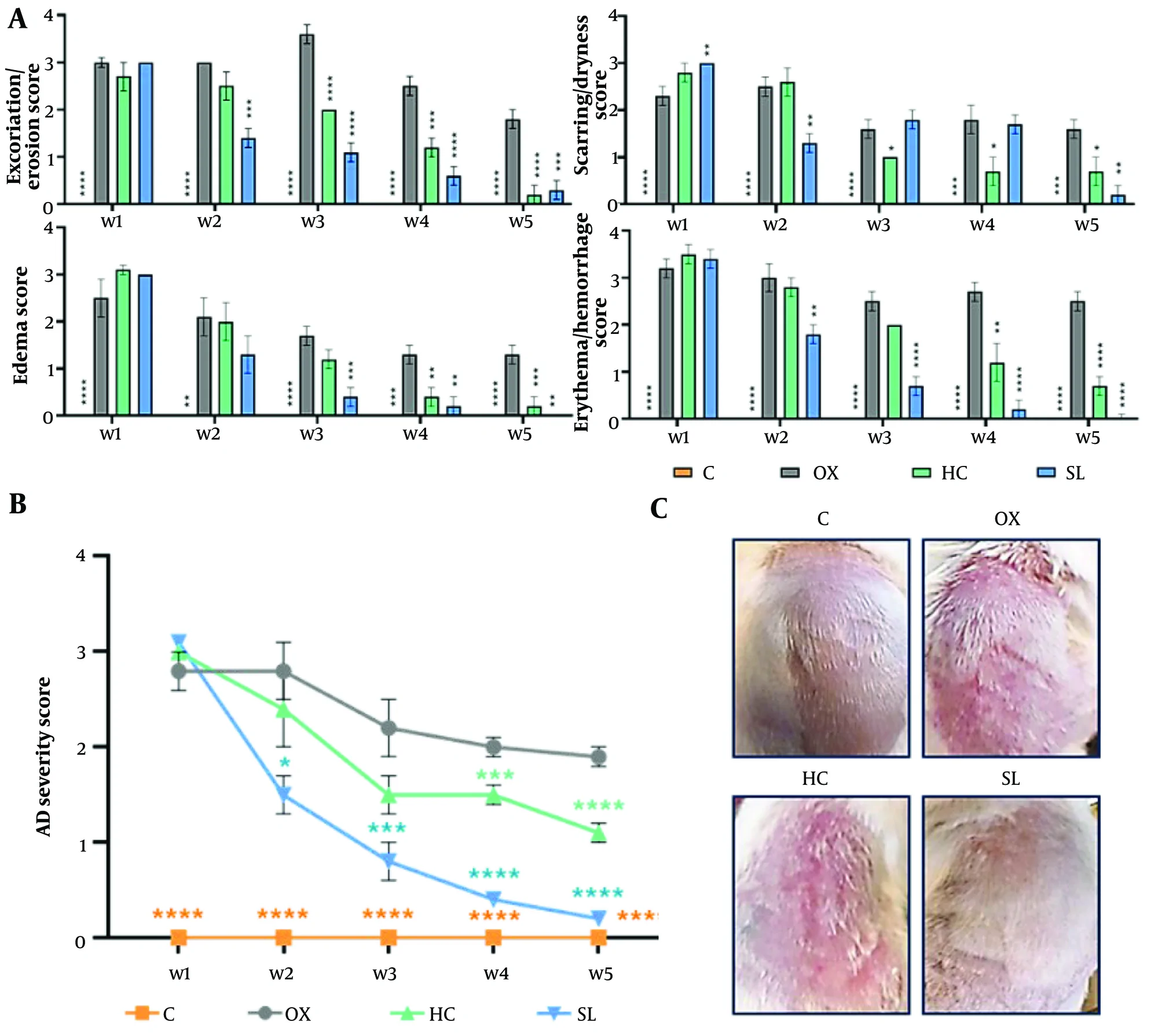
A, Graphs of excoriation/erosion, scarring/dryness, edema, and erythema/hemorrhage scores over 5 weeks. B, AD severity score over 5 weeks. Data are presented as mean ± SEM (n = 4 mice per group). *Versus OX group: * P < 0.05, ** P < 0.01, *** P < 0.001, ****P < 0.0001. C, Macroscopic skin changes in the fifth week showing that HC and SL therapies were effective in AD mice.

Data in Figure 2 indicate that the OX group had the increased excoriation/erosion, scarring/dryness, edema, and erythema/hemorrhage scores compared with the other groups at each time interval (more experimental data are presented in Figure 2A). As shown in Figure 2B, the AD severity score in the OX group was significantly increased (P < 0.0001) compared with the C group in each corresponding week. Moreover, the AD severity score was significantly decreased in the HC group in weeks 4 and 5 (P < 0.001 and P < 0.0001, respectively) and in the SL group in weeks 2 - 5 (P < 0.05, P < 0.001, P < 0.0001, and P < 0.0001, respectively) in comparison to OX. As shown in Figure 2C, the OX group exhibited severe AD symptoms after induction, whereas HC and SL treatments markedly improved the appearance of eczema.

### 4.3. Behavioral Assessments (Secondary Outcome)

**Figure 3.**
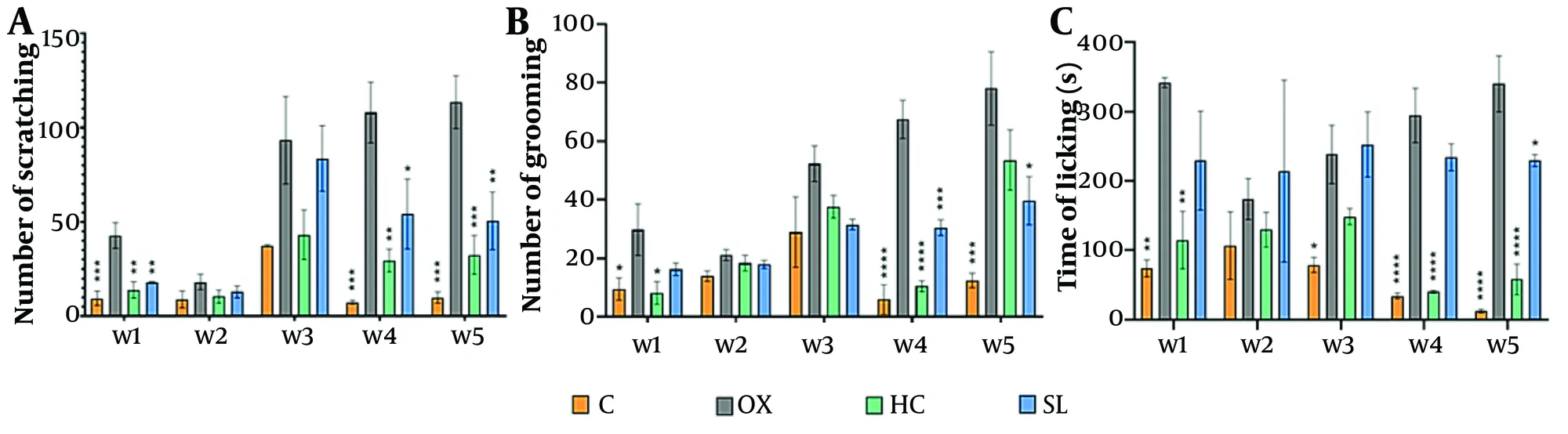
A-C, Numbers of scratching and grooming reactions and licking duration over 5 weeks of experimentation. Data are presented as mean ± SEM (n = 4 mice per group). *Versus OX group: * P < 0.05, ** P < 0.01, *** P < 0.001, **** P < 0.0001.

Animal behavior was evaluated at the end of each week. As shown in Figure 3A-C, the numbers of scratching and grooming reactions and the licking duration increased in the OX group compared with the C group at all experimental time points. Moreover, at most time points, all behavioral signs were significantly decreased (P < 0.05) in the HC- and SL-treated groups compared with the OX group. Further details of the behavioral reactions are presented in Figure 3.

### 4.4. Histopathological and IHC Analysis (Exploratory/Mechanistic Outcome)

**Figure 4.**
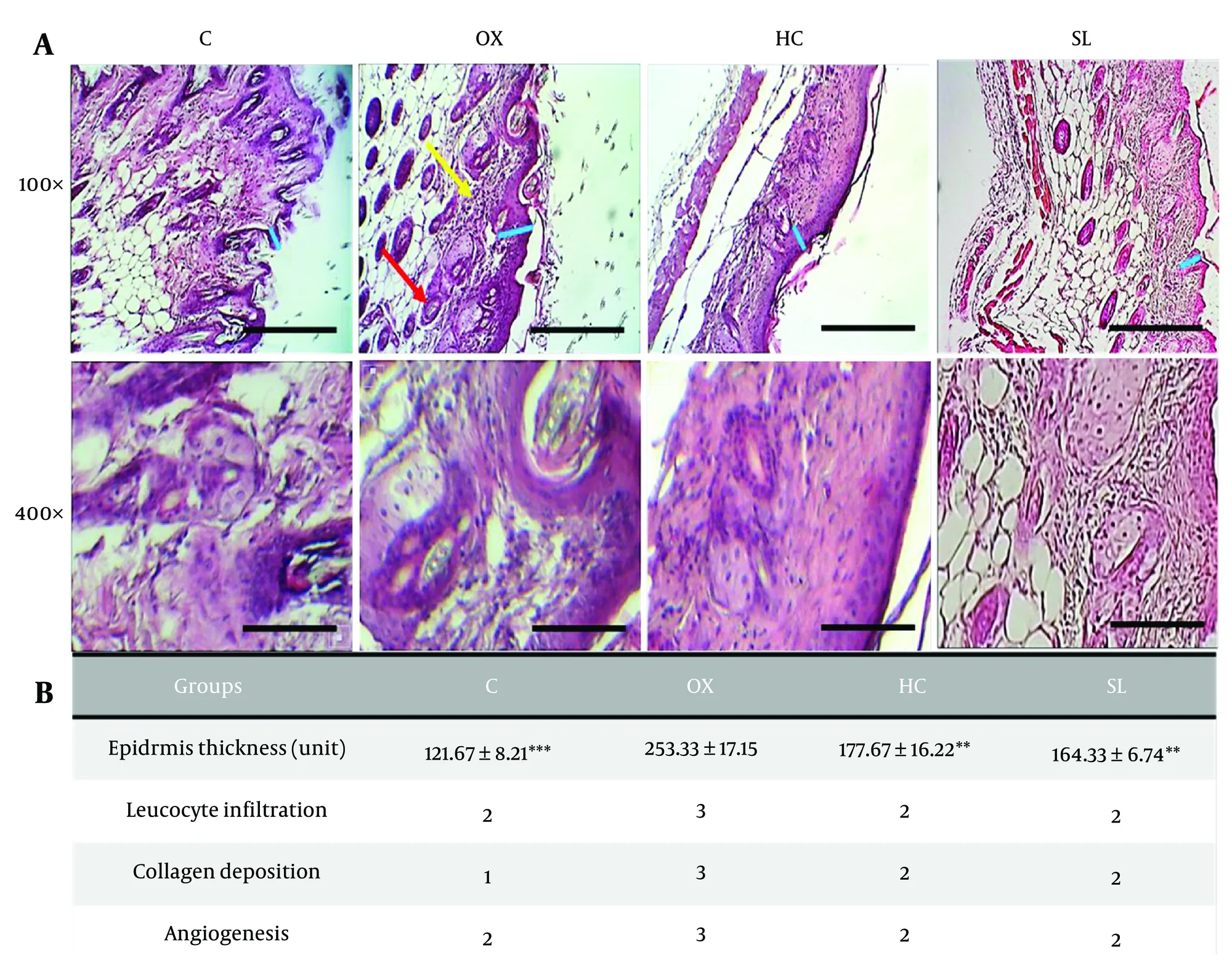
Histopathological results of experimental AD mice 1 day after the end of the fifth week. A, Microscopic skin changes with H&E staining. Blue, red, and yellow margins indicate epidermal thickness, angiogenesis, and leukocyte infiltration, respectively. B, Histopathological data, including epidermal thickness, leukocyte infiltration, collagen deposition, and angiogenesis. Data are presented as mean ± SEM (n = 4 mice per group). *Versus OX group: * P < 0.05, ** P < 0.01, *** P < 0.001.

Histopathological evaluation showed that epidermal thickness in the OX group was significantly greater than that in the C group (P < 0.001). Both SL and HC treatments significantly reduced epidermal thickness compared with that in the OX group (P < 0.01). Leukocyte infiltration increased in the OX group compared with the treated groups, but the difference was not significant (P > 0.05). Moreover, collagen deposition and angiogenesis were decreased in the SL and HC groups compared with the OX group, without significant differences (P > 0.05) (Figure 4B).

**Figure 5.**
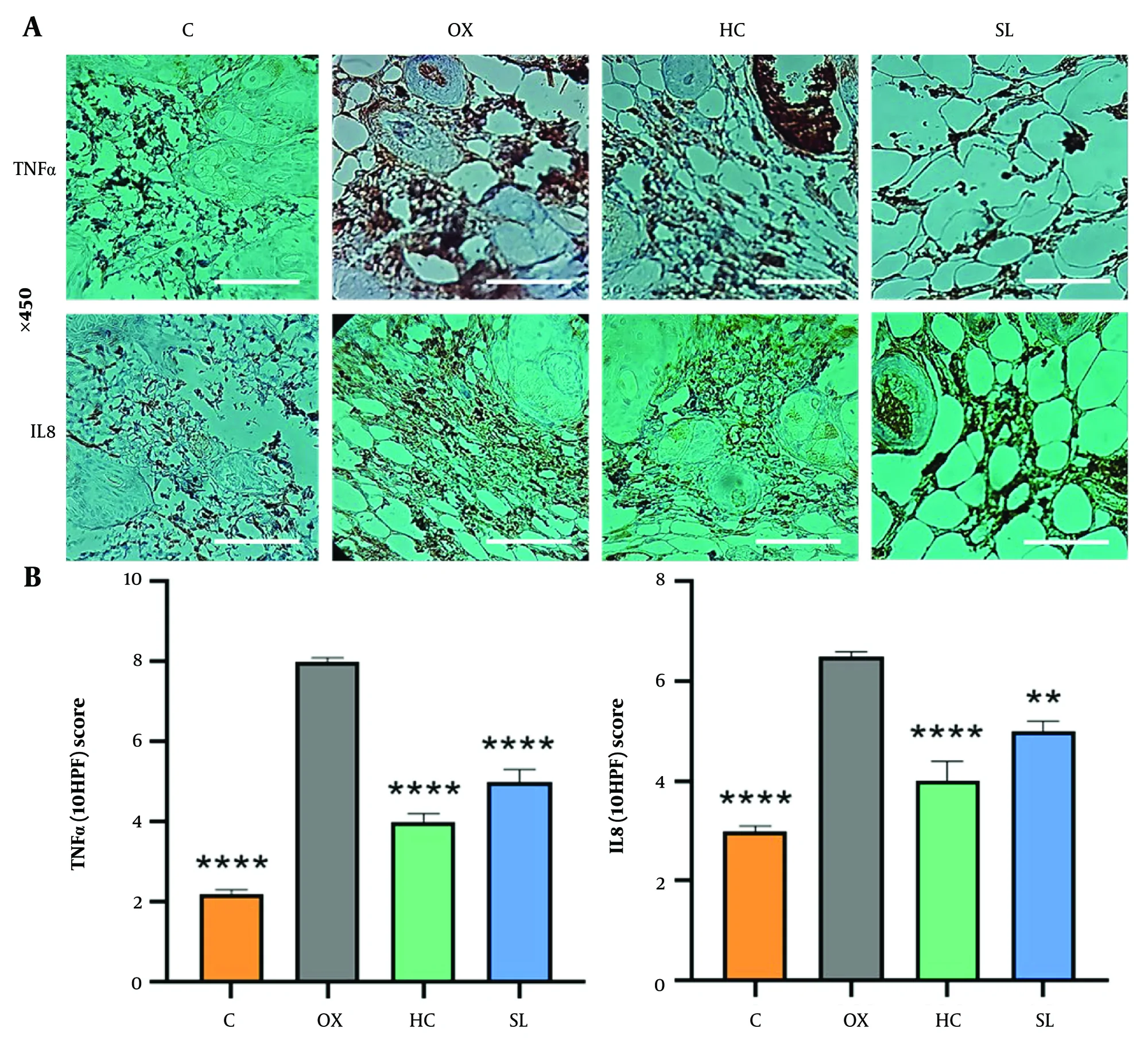
A, Microscopic skin changes with IHC staining. Brown spots indicate TNFα and IL8 cytokine expression. B, TNFα and IL8 scores. Data are presented as mean ± SEM (n = 4 mice per group). *Versus OX group: ** P < 0.01, **** P < 0.0001.

Furthermore, as shown in Figure 5, TNFα and IL8 expression was significantly higher in the OX group than in the C group (P < 0.01). Topical SL significantly reduced TNFα (P < 0.0001) and IL8 (P < 0.01) expression compared with the OX group. Moreover, HC treatment significantly decreased both cytokines compared with the OX control (P < 0.0001) (Figure 5A and B).

## 5. Discussion

In this study, we evaluated the anti-eczematous effects of topical SL compared with HC, the standard drug for AD, in mice subjected to OX. Oxazolone was applied topically to induce contact dermatitis in the animals. Our experimental data showed that OX induced significant signs of AD in the dorsal skin of mice during the 5-week study.

Moreover, the results showed that topical SL significantly alleviated the AD severity score, itching and licking behaviors, and histopathological markers in AD mice. An important property of SL liposomes is their ability to cross the stratum corneum, which is related to the high flexibility of lecithin liposomes in traversing tissue barriers, particularly in skin diseases. Within the size range used in our experiment, liposomes show highly efficient penetration and retention in tissue layers and exert anti-inflammatory and therapeutic effects in wound healing (15, 18, 27).

Itching is the predominant symptom of inflammatory AD and significantly impairs the quality of life of affected individuals. The sensation of itching triggers the scratch reflex and increases the compulsion to scratch, leading to aggravation of skin lesions (28), with the potential for secondary infections and sleep disturbances (29). Consequently, alleviation of pruritus is considered a fundamental strategy for managing the progression of skin lesions in individuals with eczema (28). Corticosteroids such as HC are a mainstay of treatment for eczema but can also cause major adverse effects (7).

Interestingly, topical SL significantly modulated collagen deposition and reduced leukocyte infiltration in the skin tissues of AD mice compared with HC. Fibrocystic accumulation during skin fibrogenesis can also lead to the production of inflammatory cytokines that induce collagen at the lesion site (30). Eczematous lesions are characterized by epidermal hyperplasia and increased dermal inflammatory-cell infiltration, including monocytes, macrophages, mast cells, basophils, eosinophils, and neutrophils, on histopathological analysis (31, 32).

In this study, angiogenesis was markedly reduced in SL-treated mice compared with the untreated and HC groups. Angiogenesis usually occurs as a physiological process and may also occur during inflammatory diseases (33). Meanwhile, topical SL modulated epidermal hyperplasia in AD mice to a considerable degree, which is an important feature of eczematous conditions.

Our histopathological data showed that lecithin administration has a beneficial role in reducing the development of inflammation. Certain phospholipids in lecithin, such as PS and PC, have shown anti-inflammatory and immunomodulatory effects in the body (34-36). For example, PC, the main phospholipid in lecithin (11), is a target of MQ phagocytosis and modulates the proliferation of activated lymphocytes (17). In vitro and in vivo studies have indicated that lecithin phospholipids lead to a significant decrease in lymphocyte proliferation and interleukin-2 production in response to concanavalin A (17). Moreover, in a study by Nasab et al. (15), topical administration of soy and egg lecithins showed a wound-healing effect in animals associated with anti-inflammatory and antioxidative effects. Phosphatidylserine, another phospholipid present in SL, plays a key role in anti-inflammatory and immunomodulatory effects in both physiological and pathological states. PS administration activates phagocytosis through apoptotic mimicry and triggers "eat me" signaling in immune cells (37, 38). This phenomenon leads to depletion of proinflammatory cytokines such as TNFα, IL1, and IL6 while enhancing the production of anti-inflammatory cytokines such as TGFβ (38).

In the present study, the proinflammatory cytokines TNFα and IL8 were analyzed in AD mice using IHC staining after 36 days of treatment with topical SL. TNFα (2, 39) and IL8 (9, 40) are cytokines involved in the development of contact dermatitis. IL8 is a nonspecific mediator with mitogenic activity in keratinocytes that induces inflammatory or immunogenic factors after stimulation with contact sensitizers and irritants (40). Furthermore, upregulation of TNFα in conjunction with other inflammatory cytokines plays a significant role in the development of epidermal abnormalities and skin-barrier dysfunction. This process ultimately leads to skin thickening and scale formation (2, 4).

### 5.1. Study Limitations

Further investigation of additional hallmarks or mechanisms is essential to substantiate the anti-AD properties of SL. Animal studies have limitations that can prevent precise investigation of dermatitis. For instance, ethical concerns in the conduct of animal studies, the inability to describe itching in a human-like manner, and differences in animal skin physiology can complicate a full understanding of mechanisms using current animal models. Therefore, our findings should be confirmed in further preclinical and clinical trials before generalization to human patients.

### 5.2. Conclusions

Our findings suggest that topical application of SL significantly improved AD severity and reduced proinflammatory cytokines, including TNFα and IL8, in an OX-induced mouse model of AD. This alleviation could lead to restoration of lipid homeostasis in the skin barrier at the lesion site and improvement of dermatitis symptoms. These results support further preclinical validation, including dose optimization, safety assessment, and mechanistic studies, before clinical translation is considered. However, further investigation of additional hallmarks or mechanisms will be essential to substantiate the anti-AD properties of SL.

## Data Availability

The dataset presented in the study is available on request from the corresponding author during submission or after publication.
